# Bio-Based Pyrrole Compounds Containing Sulfur Atoms as Coupling Agents of Carbon Black with Unsaturated Elastomers

**DOI:** 10.3390/nano13202761

**Published:** 2023-10-14

**Authors:** Gea Prioglio, Simone Naddeo, Ulrich Giese, Vincenzina Barbera, Maurizio Galimberti

**Affiliations:** 1 Department of Chemistry, Materials and Chemical Engineering Giulio Natta, Politecnico di Milano, Via Mancinelli 7, 20131 Milano, Italy; gea.prioglio@polimi.it (G.P.); simone.naddeo@polimi.it (S.N.); 2 Deutsches Institut für Kautschuktechnologie e. V., Eupener Straße 33, 30519 Hannover, Germany; ulrich.giese@dikautschuk.de

**Keywords:** sulfur-based pyrroles, sp^2^ carbon allotropes, poly(1,4-*cis*-isoprene), reduction in CO_2_ emission

## Abstract

In this work, the hysteresis of elastomer composites suitable for tire compounds was reduced by using CB functionalized with pyrrole compounds containing sulfur-based functional groups reactive with the elastomer chains. CB was functionalized with bio-based pyrrole compounds: 2-(2,5-dimethyl-1*H*-pyrrol-1-yl)ethane-1-thiol (SHP) and 1,2-bis(2-(2,5-dimethyl-1*H*-pyr-rol-1-yl)ethyl)disulfide (SSP), bearing an -SH and an -SS- functional group, respectively. SHP and SSP were synthesized via a one-pot two-step synthesis, with yields higher than 70%, starting from biosourced chemicals as follows: 2,5-hexanedione from 2,5-dimethylfuran, cysteine and cysteamine. The functionalization of CB was carried out by mixing the CB with PyC and heating, with quantitative yields ranging from 92 to 97%. Thus, the whole functionalization process was characterized by a high carbon efficiency. The formation of the covalent bond between SHP, SSP and CB, in line with the prior art of such a functionalization technology, was proven by means of extraction and TGA analyses. The reactivity of the sulfur-based functional groups with unsaturated polymer chains was demonstrated by using squalene as the model compound. Poly(styrene-co-butadiene) from solution anionic polymerization and poly(1,4-cis-isoprene) from Hevea Brasiliensis were the elastomers employed for the preparation of the composites, which were crosslinked with a sulfur-based system. Pristine CB was partially replaced with CB/SHP (33%) and CB/SSP (33% and 66%). The PyC resulted in better curing efficiency, an increase in the dynamic rigidity of approximately 20% and a reduction in the hysteresis of approximately 10% at 70 °C, as well as similar/better ultimate tensile properties. The best results were achieved with a 66% replacement of CB with CB/SSP. This new family of reactive carbon blacks paves the way for a new generation of ‘green tires’, reinforced by a CB reactive with the polymer chains, which provides high mechanical properties and low rolling resistance. Such a reactive CB eliminates the use of silica, and thus the ethanol emission resulting from the condensation of silane is used as a coupling agent. In addition, CB-based tires are characterized by a higher mileage, at a moment in which the reduction in tire wear has become a primary concern.

## 1. Introduction

To move people and things is a fundamental need of humanity. The United Nations (UN) already highlighted the importance of transport for sustainable development [1] at the 1992 United Nations’ Earth Summit. Indeed, the 2030 Agenda for Sustainable Development presents sustainable transport among the Sustainable Development Goals. The numbers regarding mobility call for urgent actions. The private car traffic and global freight volumes will increase by 50% and 70% compared with 2015, according to the Global Mobility Report 2017 of the UN [2]. The number of cars circulating will be approximately 2.4 billion.

Tires play a fundamental role in pursuing the objective of sustainable mobility, as they can make a great contribution to the reduction in the so-called rolling resistance, which means the energy consumed per unit distance [3]. The global tire market is forecast to produce over 2.7 billion tires by 2026 [4]. Unsaturated rubbers are the most useful polymers for the preparation of elastomeric composites. Indeed, they give entropic elasticity to the composites, whereas the reinforcing fillers promote static and dynamic-mechanical reinforcement [5]. The reinforcing fillers should keep as the hysteresis as low as possible, hence the dissipation of energy in the composites. To achieve this goal, silica [5,6] is the reinforcing filler of election, with respect to carbon black (CB) [5,7]. Both silica and CB are nanostructured fillers, with nanosized primary particles and voids in the particles’ aggregates. These features allow for the establishment of a large elastomer/filler interfacial area and the occluded elastomers’ chains, thus promoting mechanical reinforcement. The uniqueness of silica is the presence on its surface of reactive silanols, which react with sulfur-based silanes, such as bis(triethoxysilylpropyl)tetrasulfide, as coupling agents between silica and the elastomer chains. The covalent bond between silica and the elastomer is the molecular basis for achieving remarkable mechanical reinforcement and low dissipation of energy. Research is continuously performed to further improve the properties of silica-based elastomer composites [8,9]. 

Hence, a chemical bond between CB and the elastomers’ chains would be highly desirable, but the CB grades available on the market, which are essentially from furnace technology [7], do not have chemical reactivity in their pristine state. To be chemically reactive, they should be functionalized. In the prior art, the functionalization of carbon black has been characterized by experimental conditions that were harsh and/or difficult to scale up. Moreover, dangerous and even toxic reagents were used. The introduction of oxygen atoms on the CB surface has been obtained with ozone [7,10,11,12,13,14], potassium permanganate [15], potassium dichromate [15], hydrogen peroxide [16,17], nitric acid [15,18], and plasma [19,20,21,22,23]. Nitrogen atoms were introduced through the reaction with diazomethane [24], ammonia [25,26,27], triazole [28] and sulfur atoms, which could be reactive with the elastomer chains through the vulcanization chemistry with sulfur vapor [29,30], hydrogen sulfide [31,32,33,34,35], sulfur dioxide [36,37], carbon disulfide [38] and a polysulfide [39].

**Figure 1:**
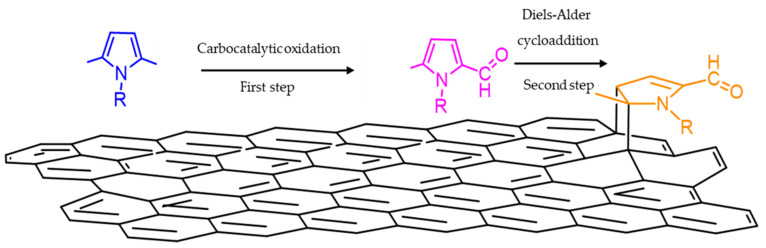
Domino reaction of pyrrole compounds with graphene layers.

Instead, the functionalization reaction should ideally comply with the principles of green and sustainable chemistry [40,41,42]. Moreover, the functional group should be reactive with the polymer chains without the need for a coupling agent. The functionalization of sp^2^ carbon allotropes with pyrrole compounds (PyC) was presented by some of the authors [43,44]. Functional groups were covalently bound to the surface of carbon black [43,44,45], nanosized graphite [46], and carbon nanotubes [47] by first mixing them with a pyrrole compound, donating either mechanical or thermal energy. To explain these findings, the domino mechanism, shown in Figure 1, was elaborated [48]. The pyrrole compound experiences oxidation in the benzylic position(s), promoted by the carbon substrate. With electron withdrawing substituents in alpha position(s), the pyrrole compound is activated for the cycloaddition reaction with the carbon material, acting as a dienophile. A covalent adduct is formed.

The objectives of the work reported here were: to synthesize the pyrrole compounds by using only bio-based reagents; to functionalize CB with high atom efficiency and a low E factor and to introduce functional groups on the carbon surface that can react with the elastomer chains in the elastomer composite without the need of a coupling agent. The further objective was to obtain an elastomer composite based on functionalized carbon black with a higher dynamic modulus and lower hysteresis with respect to the reference compound based on pristine CB.

**Figure 2:**
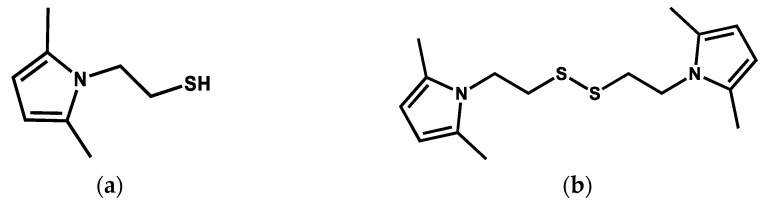
(**a**) 2-(2,5-dimethyl-1*H*-pyrrol-1-yl)ethane-1-thiol (SHP) and (**b**) 1,2-bis(2-(2,5-dimethyl-1*H*-pyrrol-1-yl)ethyl)disulfide (SSP).

To achieve these objectives, two pyrrole compounds containing sulfur atoms were synthesized: 2-(2,5-dimethyl-1*H*-pyrrol-1-yl)ethane-1-thiol (SHP) and 1,2-bis(2-(2,5-dimethyl-1*H*-pyrrol-1-yl)ethyl)disulfide (SSP), shown in Figure 2a,b, respectively.

The reasoning behind the research activity was the choice of SHP and SSP for functionalizing CB: the pyrrole ring was expected to form a covalent bond with CB, in line with the prior art [43,44,45,46,47,48], and the sulfur-containing functional groups were expected to react with the unsaturated elastomers. The reaction of SH with a terminal C=C double bond is known as thiol-ene click chemistry, which is expected to be simple, highly efficient, and without by-products [49]. A mercaptosilane such as 3-(triethoxysilyl)-1-propanethiol was used to promote the chemical bond between silica and unsaturated elastomers, elaborating on the reactivity of the thiol group with both terminal and internal C=C double bonds [50]. The pyrrole compounds were prepared, moving from cysteine and from cysteamine, and the disulfide from cysteine. Cysteine was reported to give rise to an ultrafast thiol-ene reaction [51].

In the present work, both terminal and internal double bonds were in the elastomers, which were poly(styrene-co-butadiene) from anionic solution polymerization (S-SBR) and poly(1,4-*cis*-isoprene) from *Hevea brasiliensis* (natural rubber, NR). An appropriate combination of these two elastomers leads to formulating recipes for tire tread compounds. SHP and SSP were prepared through the reaction of 2,5-hexanedione (HD) with cysteamine and cystamine, respectively, using biosourced starting materials. Indeed, HD was synthesized from a biomass derivative such as dimethyl furan. The PyC was prepared through a two-step one-pot process [52], preparing first HD through the acid-catalyzed opening of the furan ring and then the pyrrole derivative through the reaction with the sulfurated primary amine. The characterization of the PyC was performed using ^1^H-NMR and GC–MS analysis. The functionalization of CB with SHP and SSP was carried out by mixing CB with the sulfurated pyrrole compounds, providing thermal energy [43,44,45]. The CB/SHP and CB/SSP adducts were characterized by means of solvent extraction and thermogravimetric analysis (TGA). CB/SHP and CB/SSP were used in the elastomer composites in partial replacement of CB: 33% (SHP and SSP) and 66% (SSP). SHP was also used as an ingredient in the composites to compare its behavior with that of CB/SHP. The elastomer composites were prepared by melt blending in an internal mixer and were crosslinked with a sulfur-based system. They were characterized by studying the crosslinking reaction, determining the dynamic mechanical properties by applying sinusoidal stress in the shear and in the axial modes, and determining the tensile properties through quasi-static measurements. Filler dispersion analysis was performed. The reaction of SHP and SSP with unsaturated polymer chains was investigated by using (6E,10E,14E,18E)-2,6,10,15,19,23-hexamethyltetracosa-2,6,10,14,18,22-hexaene (squalene) as a model compound. Data on the use of CB/SHP and CB/SSP in elastomer composites have been reported far only in the patent literature [53].

## 2. Materials and Methods

### 2.1. Materials

For the synthesis of pyrrole compounds. 2,5-dimethylfuran (MW = 96.13 g/mol), cysteamine hydrochloride (MW = 113.61 g/mol) (purity ≥ 97%), cystamine dihydrochloride (MW = 225.2 g/mol) (purity ≥ 96%), sodium acetate trihydrate (MW = 136.08 g/mol) (purity ≥ 99%), and dichloromethane (MW = 84.93 g/mol) (purity ≥ 99.5%) were purchased from Sigma-Aldrich.

For the synthesis of CB/PyC adducts. The pyrrole compounds SHP and SSP were synthesized in the present work, as described below. CBN234 with a carbon content not lower than 98 wt.%, surface area 113 m^2^/g, average elemental particle size 27 nm and average aggregate size 152 nm was from Cabot Corporation, Ravenna, Italy.

For the reactions of SHP and SSP with squalene. 2,6,10,15,19,23-Hexamethyl-2,6,10,14,18,22-tetracosahexaene (squalene, MW = 410.72 g/mol) was from Sigma Aldrich. Zinc oxide (ZnO, from Flexsys, St. Louis, MO, USA), stearic acid (from Sogis, Cremona, Italy), N-tert-butyl-2-benzothiazyl sulfonamide (TBBS, from Flexsys, St. Louis, MO, USA) and Sulfur (S, from Solfotecnica, Ravenna, Italy).

For the preparation of elastomer composites. Solution styrene-butadiene synthetic rubber (S-SBR) from TRINSEO, Milan, Italy (commercial name: SPRINTAN^TM^ SLR-4630), extended with 37.5 parts of TDAE oil, with a styrene content of 25%, a vinyl content of 61.7% and 55 as Mooney viscosity (ML(1 + 4)100 °C) and a glass transition temperature (Tg) of −28 °C. Poly(1,4-cis-isoprene) from *Hevea brasiliensis* (NR) (STR/SMR/SIR 20, RSS3, from Von Bundit Co., Mueang, Phuket, Thailand), Carbon black (CB) N 234 was purchased from Cabot Corporation. The data sheet reports a carbon content not lower than 98 wt.%, surface area 113 m^2^/g, average elemental particle size 27 nm and average aggregate size 152 nm, zinc oxide (ZnO, from Flexsys, St. Louis, MO, USA), stearic acid (from Sogis), (1,3-dimethyl butyl)-N′-phenyl-p-phenylenediamine (6PPD, from Crompton), (N-tert-butyl-2-benzothiazyl sulfonamide (TBBS, from Flexsys), Sulfur (S, from Solfotecnica, Ravenna, Italy), TDAE (treated distillate aromatic extracted) oil Vivatec 500 (Hansen & Rosenthal KG, Hamburg, Germany), RIOWAX BM01 (wax from SER WAX INDUSTRY, Santena, Torino, Italy), and *N*-(cyclohexylthio) phthalimide (from Nocil Limited).

All the chemicals were used without further purification.

### 2.2. Preparation of Pyrrole Compounds, Adducts and Elastomer Composites

#### 2.2.1. Synthesis of 2-(2,5-Dimethyl-1*H*-pyrrol-1-yl)ethane-1-thiol (Thiol Pyrrole, SHP)

SHP was prepared via a one-pot two-step synthesis [52] exploiting the chemical reactivity of 2,5-dimethylfuran.

In particular, the first step involved the formation of 2,5-hexanedione starting from 2,5-dimethylfuran. Then, cysteamine hydrochloride and sodium acetate trihydrate were added to the solution of 2,5-hexanedione. No purification steps occurred between first and second step.

*First step, synthesis of 2,5-hexanedione starting from 2,5-dimethylfuran.* Water (0.85 mL, 47.0 mmol), sulfuric acid (1.88 mmol, 4 mol%, 10 drops) and 2,5-dimethylfuran (5.0 mL, 47.0 mmol) were introduced, in this order, in a round-bottomed flask, at room temperature under magnetic stirring. The temperature was raised to 50 °C and the reaction was left under stirring for 24 h at 400 rpm. 2,5-hexanedione was obtained as a dark brown liquid (95% yield).^1^H-NMR (*d*_6_-DMSO), δ (ppm): 2.61 (s, 4H, CH_2_), 2.09 (s, 6H, CH_3_).

*Second step, synthesis of SHP*: 2,5-hexanedione, obtained from the first step (44.65 mmol), was stirred with sodium acetate trihydrate (4.46 mmol, 607.6 mg 0.1 eq respect to 2,5-hexanedione) at room temperature for 1 h. In another round-bottomed flask, equipped with magnetic stirrer, sodium acetate trihydrate (6.075 g, 44.65 mmol) and cysteamine hydrochloride (5.072 g, 44.65 mmol) were introduced. The mixture was stirred at room temperature to obtain a homogeneous solution. Then, the solution of 2,5-hexanedione and sodium acetate trihydrate was added to the solution of cysteamine hydrochloride dropwise. The resulting solution was left under stirring at 400 rpm, at room temperature for 4 h. Then, the reaction mixture was quenched under stirring with a saturated solution of NaHCO_3_, until the end of the effervescence. The obtained organic phase was extracted with dichloromethane (3 × 50 mL) and anhydrified with Na_2_SO_4_. The solvent was evaporated under reduced pressure. SHP was obtained as a dark brown liquid with a yield of 73%.

^1^H-NMR (CDCl_3_), δ (ppm): 5.78, (s, 2H, H_β_) 3.95–3.91 (m, 2H, -CH_2_) 2.75–2.66 (m, 2H, -CH_2_), 2.24 (s, 6H, -CH_3_), 1.36 (t, 1H, -SH). GC–Mass analysis retention time = 14.50 min, molecular peak = 155 *m*/*z*.

#### 2.2.2. Synthesis of 1,2-Bis(2-(2,5-dimethyl-1*H*-pyrrol-1-yl)ethyl)disulfide (Disulfide Pyrrole, SSP)

SSP was synthesized via one-pot two-step synthesis [52], exploiting the chemical reactivity of 2,5-dimethylfuran with water and sulfuric acid for the preparation of 2,5-hexanedione. Then, cystamine dihydrochloride and sodium acetate trihydrate were added to the solution of 2,5-hexanedione. Purification was not performed of the products of both step 1 and step 2.

*First step, synthesis of 2,5-hexanedione starting from 2,5-dimethylfuran*: The synthetic procedure adopted for the first step was the same applied for the synthesis of SHP. 2,5-hexanedione was obtained as a dark brown liquid with a yield up to 95% (44.65 mmol). 2,5-hexanedione was obtained, without any further purifications, as a dark brown liquid. ^1^H-NMR analysis confirmed 2,5-hexanedione was obtained with a yield up to 95%. The conversion was estimated by ^1^H-NMR spectrum.

*Second step, synthesis of SSP*: Sodium acetate trihydrate were fed to the round-bottomed flask containing 2,5-hexanedione (0.1 eq. with respect to 2,5-hexanedione: 4.46 mmol, 607.6 mg). The mixture was stirred at 100 °C for 1 h. Sodium acetate trihydrate (6.075 g, 44.65 mmol) and cystamine dihydrochloride (5.072 g, 22.32 mmol) were introduced in another round-bottomed flask and left under magnetic stirring at 100 °C for 1 h, to obtain a homogeneous solution. Then, the solution of 2,5-hexanedione obtained from the first step was added to the solution of cystamine dihydrochloride dropwise. The resulting solution was stirred at 100 °C for 2 h, 400 rpm. After two hours, the final reaction mixture was quenched with a saturated solution of NaHCO_3_ until the end of the effervescence. Ethyl acetate (3 × 50 mL) was used for the extraction of the organic phase, which was then anhydrified with Na_2_SO_4_. Removal of the solvent occurred under reduced pressure.

A dark orange solid was obtained (78% yield).^1^H-NMR (CDCl_3_), δ (ppm): 5.78 (s, 4H, H_β_), 4.12–4.02 (m, 4H, -CH_2_), 2.87–2.83 (m, 4H, -CH_2_), 2.25 (s, 12H, -CH_3_).

GC–Mass: retention time = 25.76 min; molecular peak = 308 *m*/*z*.

ESI-Mass: [M + Na]^+^: Calculated exact mass = 331.13 *m*/*z*; found exact mass = 331.2 *m*/*z*.

#### 2.2.3. Preparation of Adducts of CB with Thiol Pyrrole (SHP) and Disulfide Pyrrole (SSP)

An amount of 10 parts of pyrrole compound for one hundred parts of carbon (phc) was used at the beginning of each functionalization reaction. 20 g of CB were dispersed in 50 mL of acetone in a 250 mL round-bottomed flask and were then sonicated for 10 min, by using a 2 L ultrasonic bath 260 W (SONICA, SOLTEC Srl, Milan, Italy). Then, a solution of 2 g of either thiol pyrrole (SHP) or disulfide pyrrole (SSP) in 15 mL of acetone was added to the flask. The pyrrole compound thus corresponds to 10 parts per hundred parts of carbon black. The suspension was sonicated for another 10 min. At the end of sonication the solvent was removed by using a rotavapor, at reduced pressure. The powder was transferred in a two-neck round-bottomed flask, volume 250 mL. Air flux was introduced inside the flask from one neck while an air condenser with sintered septum was put on the other neck. The temperature of the oil bath was increased up to 180 °C. The mixture was left under stirring (300 rpm) for 2 h. Extraction with Soxlet apparatus was performed on the collected powder to remove the unreacted pyrrole compound (PyC). The collected powder was dried at room temperature for 24 h and was observed to be free flowing.

#### 2.2.4. Preparation of Elastomer Composites

The recipes of the elastomer composites are reported in Table 1. The CB content was kept constant in all the composites. SPRINTAN SLR-4630 from Trinseo was selected as the S-SBR grade. CB N234 from Cabot was selected as the CB grade. A composite with SHP added as an ingredient, without forming the CB/SHP adduct ex ante, was also prepared. The amount of SHP was slightly lower than the amount of SHP in the composites with CB/SHP. Results obtained from the characterization of the CB + SHP composite are discussed in the present work, by assuming that the slight difference in the SHP content does not affect the reliability of the discussion.

**Figure 3:**
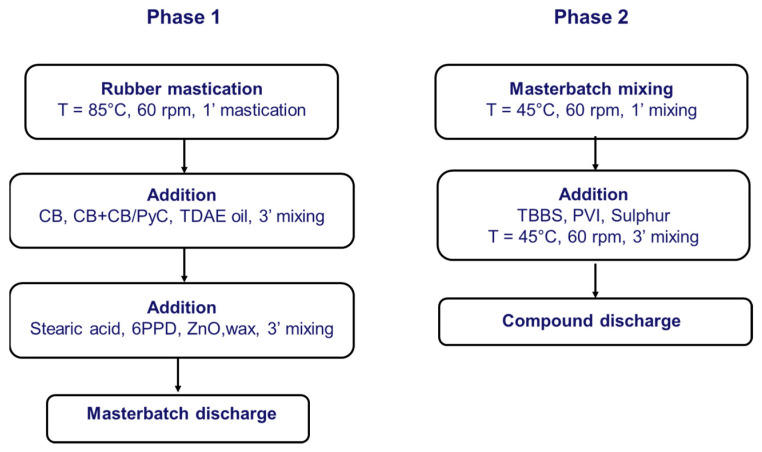
Block diagram of compound preparation procedure.

Rubber composites have been prepared by following the procedure reported in Figure 3. Each composite was prepared using a Brabender^®^ internal mixer with a 55 cc chamber (Brabender^®^ PL-2000 Plasti-Corder Torque Rheometer, Brabender GmbH & Co. KG, Duisburg, Germany). S-SBR and NR were fed into a Brabender^®^ internal mixer and masticated for 60 s at 85 °C, 60 rpm. Then, CB or CB and the CB-PyC adduct alongside TDAE oil were added, and another 3 min of mastication were performed. Then, stearic acid, zinc oxide, 6PPD and the wax were added, 3 min of mastication occurred, and the compound was discharged. Finally, masterbatch was reloaded in the mixing chamber at 45 °C, masticated for 60 s and then TBBS, PVI and sulfur were added. After 3 min of mixing, the final elastomeric composites was unloaded.

Homogenization of the composites was performed by passing them 5 times on a two-roll mill, at 50 °C, with the front and the back roll rotating at 30 rpm and 38 rpm, respectively and 1 cm as the nip between the rolls.

### 2.3. Reaction of SHP and SSP with Squalene

The reactions of SHP and SSP were performed with squalene, as model com pound of the unsaturated elastomer.

#### 2.3.1. Reaction of SHP with Squalene

The reaction between SHP and squalene was carried out in neat conditions by using a 100 mL round-bottomed flask mounted on a hot oil bath and equipped with a magnetic stirrer, a water condenser and a thermocouple. Squalene was introduced in the flask and kept under stirring. After 1 min of stirring at 200 rpm SHP was added. A stoichiometric ratio of 1:1 between SHP and squalene was employed. The reaction was performed maintaining the mixture at 150 °C, 200 rpm for 1 h. A dark yellow liquid was obtained.

#### 2.3.2. Reactivity of SHP with Squalene in the Presence of Vulcanizing Agents

The reactivity of SHP with squalene in the presence vulcanizing agents was performed by introducing squalene, stearic acid, zinc oxide, TBBS and sulfur in a 100 mL round-bottomed flask at 150 °C, following the recipe reported in Table S2 in the Supplementary Material. The flask was equipped with a water condenser, a magnetic stirrer and a hot oil bath. After 1 min of stirring at 200 rpm, SHP was added to the flask in an amount stoichiometrically equivalent to squalene. The reaction mixture was then kept at 150 °C and 200 rpm for 1 h. A brown viscous liquid was obtained.

### 2.4. Characterization of Low-Molar-Mass Chemicals: Pyrrole Compounds

#### 2.4.1. Nuclear Magnetic Resonance Spectroscopy (NMR)

NMR spectra were registered on a Bruker 400 MHz instrument Billerica, MA, USA (100 MHz) at 298 K. Chemical shifts were reported in ppm with the solvent residual peak as the internal standard (DMSO-*d*_6_: δH = 2.50 ppm; CDCl_3_: δH = 7.26 ppm).

#### 2.4.2. Gas Chromatography–Mass Spectrometry (GC–MS)

Electrospray ionization (ESI) with a Bruker Esquire 3000 plus ion-trap mass spectrometer instrument equipped with an ESI Ion Trap LC/MSn System Mass was used to perform the analyses.

The instrument for GC–MS analysis was an Agilent 5973 network mass selective detector, Milano, Italy, with a 6890 Series GC system mass spectrometer, Milano, Italy. The column used for all analyses was a J&W GC Column HP-5MS [(5%-phenyl)-methylpolysiloxane] 30 m, 0.25 mm internal diameter and 0.25 μm film thickness, Milano, Italy.

#### 2.4.3. HPLC–UV–MS Direct Injection Analyses

For HPLC–UV–MS direct injection analyses were carried out with a Velos Pro system from Thermo Fisher, Hannover, Germany. Direct injection, flow rate: 10 µL/min, APCI source (positive mode) source parameters: Vaporizer Temp. (°C) = 450, Sheath Gas Flow rate (arb) = 60, Aux Gas Flow rate (arb) = 5, Sweep Gas Flow rate (arb) = 0, Discharge Voltage (kV) = ~3.5, Capillary Temp. (°C) = 200 and S-Lens RF Level (%) = 69.

### 2.5. Characterization of CB/SHP and CB/SSP Adducts

CB-PyC adducts were characterized via the TGA technique, to evaluate the mass loss of the finale functionalized powder obtained after Soxhlet. In that way, the amount of PyC in the adduct is also calculated.

#### 2.5.1. Thermogravimetric Analysis (TGA)

A Mettler TGA SDTA/851 (Mettler Toledo, Columbus, OH, USA) was the TGA instrument. The method was the standard ISO9924-1 [54]: 5–10 mg of either CB or CB/PyC adduct experienced a heating ramp (10 °C/min) from 30 up to 300 °C, then an isotherm at 300 °C for 10 min, then a heating ramp (20 °C/min) up to 550 °C, an isotherm at 550 °C (15 min) and finally a heating ramp (10 °C/min) up to 900 °C. This temperature was kept constant until the end of the experiment. The gas in the chamber was switched from N_2_ to air, after 102.5 min from the beginning of the test. Duration of the experiment was 120 min.

#### 2.5.2. Amount of PyC in the CB/PyC Adduct (in Phc)

To calculate the amount of pyrrole compound in the adduct, expressed in parts per hundred carbon (phc), Equation (1) was used.

(1) $$phc\left(instrumental\right)=\frac{{ML(\%)\;of\;washed\;CB-PyC}_{150<T<900{}^{\circ}C}}{{ML\left(\%\right)\;of\;washed\;CB-PyC}_{T>900{}^{\circ}C}}\cdot 100$$

where ${ML(\%)\;of\;washed\;CB-PyC}_{150<T<900{}^{\circ}C}$ is the mass loss determined via TGA in the temperature range 150 < T < 900 °C, attributed to PyC in the washed CB-PyC adduct and ${ML\left(\%\right)\;of\;washed\;CB-PyC}_{T>900{}^{\circ}C}$ is the mass loss of the washed CB-PyC adduct measured above 900 °C attributed to the carbonaceous substrate and to residues, if any.

#### 2.5.3. Functionalization Yield

To calculate the functionalization yield, Equation (2) was employed.

(2) $$Functionalization\;Yield(\%)=\frac{{ML(\%)\;of\;washed\;CB-PyC}_{150<T<900{}^{\circ}C}}{{ML(\%)\;of\;unwashed\;CB-PyC}_{150<T<900{}^{\circ}C}}\cdot 100$$

where ${ML(\%)\;of\;washed\;CB-PyC}_{150<T<900{}^{\circ}C}$ are the measured mass losses between 150 °C and 900 °C of the washed adduct, ${ML\left(\%\right)\;of\;unwashed\;CB-PyC}_{150<T<900{}^{\circ}C}$ are the mass losses between 150 °C and 900 °C measured for the unpurified adduct, attributed to organic moieties.

### 2.6. Characterization of Elastomer Composites

#### 2.6.1. Crosslinking

The crosslinking reaction of rubber composites was carried out at 170 °C for 20 min. An amount of 5 g of crude rubber compound were introduced in the rheometer (a rubber process analyzer (RPA, Alpha Technologies, Hudson, OH, USA). A strain sweep was performed at low deformations (0.1–25% strain) before the crosslinking step. Then, the sample was kept at 50 °C for ten minutes and subjected to another strain sweep always at 50 °C. Subsequently, vulcanization at 170 °C was performed and the torque versus time curve, the minimum achievable torque (M_L_), the maximum achievable torque (M_H_), the time needed to have a torque equal to M_L_ + 1 (T_S1_), and the time needed to reach 90% of the maximum torque (T_90_) were measured. Acquisitions were performed with an oscillation angle of 6.98% and a frequency of 1.7 Hz.

#### 2.6.2. Dynamic-Mechanical Analyses in Shear Strain-Sweep Tests

Shear dynamic-mechanical properties were measured using a rubber process analyzer (RPA). At the crude sample was applied a strain sweep at low deformations (0.1–25% strain), then the sample was kept at 50 °C for ten minutes and subjected to another strain sweep at 50 °C before being vulcanized as described in paragraph 2.7. After 20 min at 50 °C, shear dynamic-mechanical properties were measured, applying a 0.1–25% strain sweep at a frequency of 1 Hz. The measured properties were shear storage and loss moduli (G′, G″).

#### 2.6.3. Dynamic-Mechanical Analyses in Compression

The rubber composite taken from the two-roll mill was rolled up to prepare a long cylinder. The cylinder was cut in smaller cylinders and vulcanized to give cylindrical specimens with length of approximately 25 mm and diameter of approximately 12 mm. Dynamic-mechanical measurements were performed with an Instron dynamic device in the traction-compression mode, at the following temperatures: 10, 23 and 70 °C. An axial compressive pre-strain of 25% was applied on the specimen followed by a sinusoidal axial strain sweep with an amplitude of ±3.5% with respect to the applied pre-strain, with a 100 Hz frequency. Dynamic-mechanical storage modulus (E′) and loss factor (Tan Delta) were registered.

#### 2.6.4. Quasi-Static Tensile Tests

The composite from the two-roll mill was crosslinked in a vulcanizing press to produce a 1 mm thick vulcanized plate. ISO 37 [55] specimens were used for the tests. They were dumbbell-shaped, punched from the vulcanized plate, with a length of 10 +/− 0.5 mm and a width of 4 +/− 0.2 mm (type 3). On these specimens, the tensile tests were carried out with a Zwick universal testing machine (Zwick Roell Z001, Test expert, Ulm, Germany) and an optical extensometer. Stresses at 100, 200 and 300% elongation (σ_100_, σ_200_, σ_300_, respectively) as stress at break (σ_B_), elongation at break (ε_B_) and energy at break were determined. Three measurements were performed for each sample.

#### 2.6.5. Digital Filler Dispersion Analysis

The digital image analysis of filler dispersion was carried out following three steps: (1) the vulcanized compound was microtomed at low temperature to obtain sections with thicknesses between 1 and 3 microns, (2) sections were observed in transmitted light using an optical microscope and 10 images were acquired with a digital camera for each sample, and (3) the acquired images were digitally analyzed to determine the amount of undispersed filler particles.

## 3. Results and Discussion

### 3.1. Synthesis of SHP and SSP

**Figure 4:**
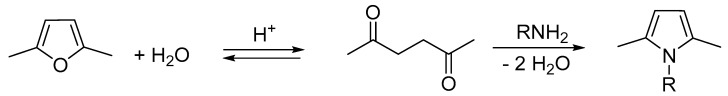
One-pot, two-step synthetic process for the preparation of SHP (R = -CH_2_-CH_2_-SH) and SSP (R = -CH_2_-CH_2_-S-S-CH_2_.CH_2_-NH_2_).

SHP and SSP were synthesized starting from 2,5-dimethylfuran as the bio-based starting material. The whole synthetic process, summarized in Figure 4, was one pot two steps.

The ring opening of DMF was promoted by a sub-stoichiometric amount of sulfuric acid and a stoichiometric amount of water, and the reaction was carried out at 50 °C for 24 h, obtaining chemically pure HD with a quantitative yield of 98% [52]. Thiol pyrrole and disulfide pyrrole were then obtained by adding the sulfurated primary amines and performing the reaction at room temperature for 4 h, in the case of SHP, or at 100 °C for 2 h, in the case of SSP. The yield was 73% and 78% for SHP and SSP, respectively.

It is worth observing the circularity of the whole process: the first step can be triggered by the water produced in the second step. The one-pot process makes its scale-up easier, and this appears of remarkable importance in consideration of the use of these substances as CB coupling agents in a large-scale application such as the one in tyre compounds.

### 3.2. Functionalization of Carbon Black with SHP or SSP

**Figure 5:**
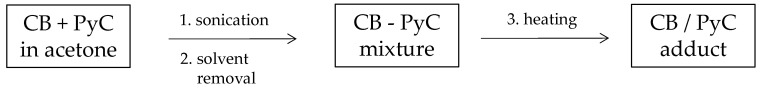
Procedure for the preparation of CB/PyC adducts, with either SHP or SSP as the PyC.

Carbon black was functionalized with either SHP or SSP as the PyC by using the so-called ‘pyrrole methodology’ [47], whose mechanism is shown in Figure 1 above. The experimental procedure, summarized in Figure 5, was used for the functionalization of CB [43,44,45], HSAG [46], and CNT [47], with other pyrrole compounds.

Details are in the experimental section. In brief, 10 parts of PyC per hundred parts of carbon (phc) were used. CB and either SHP or SSP were mixed in acetone, followed by sonication to form a homogeneous dispersion. The solvent was then removed, and a physical mixture was obtained. The temperature in the flask was increased to 150 °C after stirring the powder for 2 h under air flow. The unreacted PyC was removed by washing the adduct with acetone. In the adopted procedure, the step for the preparation of the homogeneous physical mixture requires an organic solvent (acetone) and could thus be considered a polluting step. However, in the scale-up of the process, the organic solvent could be avoided, and an apparatus such as a spray dryer [56] could reasonably be used. Functionalization occurs at high temperatures, which makes the process energy-intensive. Functionalization is made of two steps: the oxidation of the pyrrole compounds and the cycloaddition reaction. As the oxidation is needed to make the pyrrole compounds reactive with the carbon substrate, the use of pyrrole compounds with electron withdrawing groups in the alpha position(s) will be considered to adopt lower temperatures in the functionalization step.

**Figure 6:**
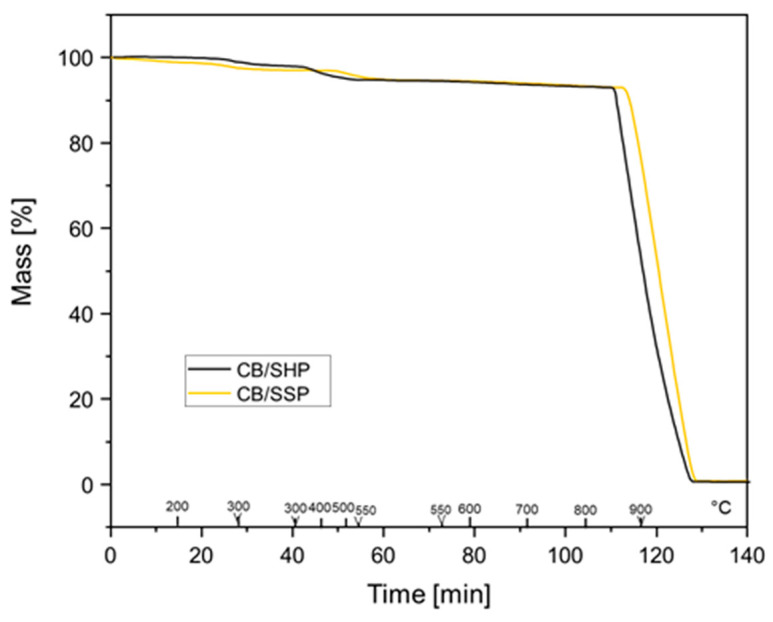
Thermogravimetric curves of purified CB N234-SHP (black), and CB N234-SSP (yellow).

CB/SHP and CB/SSP adducts were analyzed by thermogravimetric analysis (TGA). Organic mass losses of CB-PyC adducts before and after an exhaustive acetone wash were compared. Data are reported in Table 2, while thermogravimetric curves are shown in Figure 6.

In the graph in Figure 5, three main steps can be identified for the mass loss of the CB/PyC adducts: below 150 °C, between 150 °C and 900 °C, and above 900 °C. The mass loss below 150 °C can be attributed to low-molar-mass substances, such as water or acetone used for washing. The loss between 150 °C and 900 °C can be justified by the decomposition of PyC and the alkenylic defects of CB. The mass loss above 900 °C is due to the combustion of the carbon material with the oxygen fed to the chamber. As shown by the data reported in Table 2, mass losses are appreciable even after an exhaustive acetone extraction of the CB-PyC adduct. This can be taken as evidence of the formation of a stable adduct. From the TGA results, by using the values of the washed adducts, the amount of PyC in the adduct was estimated by means of Equation 1 and found to be 7 phc for SHP and 6 phc for SSP. The functionalization yield, calculated according to Equation (2), was 92% for the CB/SHP adduct and 97% for the CB/SSP adduct.

### 3.3. Preparation of the Elastomer Composites

The CB/SHP and CB/SSP adducts were used in the partial replacement of pristine CB in elastomer composites based on S-SBR and NR as the elastomer matrix. Two amounts of CB were replaced: 33% (with CB/SHP and CB/SSP) and 66% (with CB/SSP), by maintaining constant the total amount of CB. The recipes for the composites are in Table 1 in the experimental section. Mixing was performed via melt blending following the procedure reported in the experimental section. In the text below, data on the composites based on the adducts and on SHP are in the tables, whereas only curves referring to the composites with CB and CB/PyC are in Figures, to focus attention on the comparison between pristine CB and the adducts.

### 3.4. Characterization of Elastomeric Composites

#### 3.4.1. Curing

The crosslinking was performed with a sulfur-based system. Details are in the experimental part. Data of M_L_, M_H_, t_s1_, t_90_, and the curing rate are in Table 3, whereas the rheometric curves are in Figure S1 in the Supplementary Material.

Similar values of M_L_ were obtained for all the composites, as expected. In fact, M_L_ is an index of the composite viscosity, which should not be affected by the addition of a low amount of SHP or by the functionalization of CB with a low amount of PyC. The values of induction and optimum times of vulcanization were reduced, and the curing rate was increased by the replacement of CB with CB/PyC: the effect was larger with a larger amount of PyC. The functionalization of CB with sulfur atoms, in particular with a thiol group, could account for these findings. The lowest value of t_S1_ and the highest value of the curing rate were obtained with SHP: a thiol as an ingredient in the composite could indeed lead to this result. Higher values of M_H_ were achieved with both CB/SHP and CB/SSP. By comparing the data obtained with the two SSP-based composites, it appears that a larger amount of CB/SSP led to a larger M_H_ value. The extent of M_H_ is due to both the crosslinking and the filler networks. In fact, the filler network is not completely disrupted at the amplitude achieved in the shear test. In light of the similar M_L_ values, the higher value of M_H_ for the composites with CB/PyC could be attributed to more efficient vulcanization, with a higher crosslinking network density and/or shorter sulfur bridges. The lowest value of M_H_ was obtained with SHP. This indicates that an extra sulfur amount is not enough to increase the network density. It could be hypothesized that the increase of the M_H_ value in the presence of CB/SHP and CB/SSP is due to the presence of the reactive sulfur atoms as well as the sp^3^ nitrogen atoms on the CB surface (see Figure 1).

#### 3.4.2. Dynamic-Mechanical Properties in the Shear Mode

**Figure 7:**
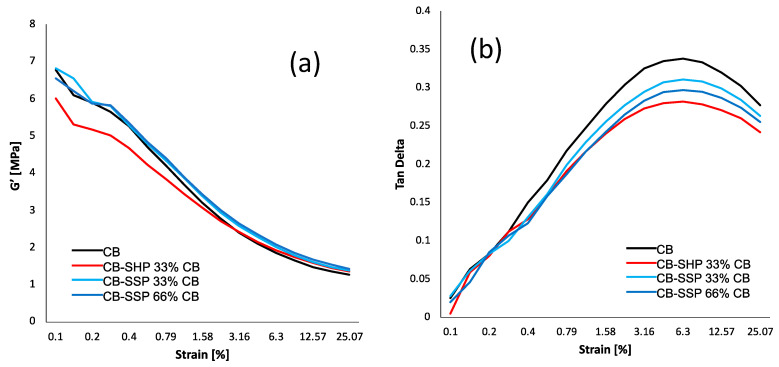
G′ vs. strain amplitude (**a**) and Tan Delta versus strain amplitude (**b**) for composites of Table 1.

Dynamic-mechanical properties were determined in the shear mode by means of strain sweep experiments, employing a strain amplitude in the range of 0.1% to 25% at a frequency of 1 Hz and a temperature of 50 °C. Details are in the experimental section. G′ and G″ moduli were measured. The graphs showing the dependence on the strain amplitude of G′ and Tan delta are in Figure 7a,b, respectively, and the data are in Table S1 in the Supplementary Material.

The extent of the filler network, formed by the interaction of the filler particles, either directly or mediated by the polymer chains, is indicated by the value of G′ at the minimum strain. The reduction in G′ with the strain amplitude, i.e., the non-linearity of the storage modulus, is the so-called Payne effect [57,58,59,60], which is due to the disruption of the filler network. The (ΔG′/G′_0_._1%_) is here taken as the normalized index of the Payne effect. Similar values were obtained with all the composites, as if the introduction of the PyC, either as an ingredient or bound to the CB surface, did not have a remarkable effect on the filler networking. However, it is worth observing the difference between the two composites with CB + SHP and CB/SHP. The former shows a much larger value of G′_0_._1%_ and of ΔG′. This finding suggests that SHP plays a different role, if it is an ingredient or it is bound to the CB surface. The values of G′ at large strain were higher, though only slightly, with the CB/PyC based composites, which also revealed lower values of G″ max. Lower values of Tan Delta were obtained in the presence of the CB/PyC adducts. These findings could suggest that the CB/PyC adducts can establish a chemical bond with the polymer chains, enhancing the large strain reinforcement. However, in consideration of the minor differences, it is wise not to stretch inferences too far before supporting them with further experimental data.

#### 3.4.3. Dynamic-Mechanical Properties from Axial Compression Tests

**Figure 8:**
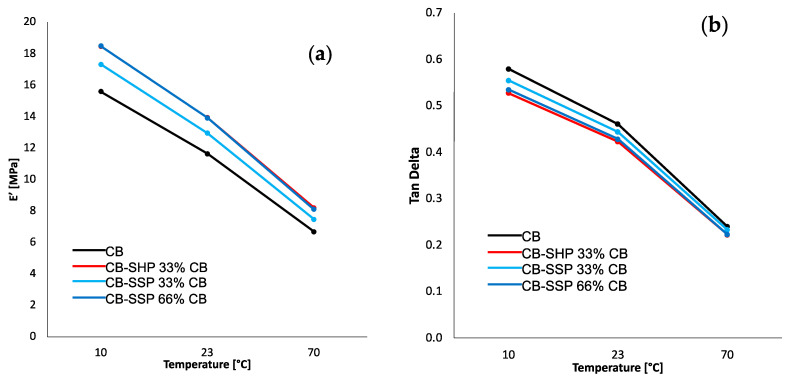
(**a**) E′ vs. temperature curves and (**b**) Tan Delta vs. temperature curves of S-SBR/NR-based composites filled with CB or CB/CB/SHP or CB/CB/SSP.

Axial dynamic mechanical analyses in compression were carried out by first applying a pre-strain of −25%, then a dynamic sinusoidal strain of 3.5% at a frequency of 100 Hz. Storage modulus (E′) and loss modulus (E″) were measured at 10 °C, 23 °C, and 70 °C. Results are reported in Table 4. The dependence of E′ and Tan Delta on the temperature is shown in Figure 8a,b, respectively.

The replacement of CB with CB/PyC led to higher dynamic rigidity and a reduction in hysteresis at all temperatures. With CB/SSP, greater values of E′ and lower values of Tan delta were obtained with a greater CB replacement. With CB + SHP, the dynamic rigidity was even lower than that of the reference composite with only CB. It is worth remembering that the amount of SHP in the CB + SHP composite was slightly higher than in the CB/SHP composite. Moreover, with SHP used as an ingredient, greater values of hysteresis were obtained. These results support the comment reported above: the effect of SHP is much different when it is an ingredient or it is bound to the CB surface. More than that, they appear to be in line with what has been reported so far. SHP and SSP behave as coupling agents for CB when they are in the CB/SHP and CB/SSP adducts. It is worth observing that the best results, the increase of more than 20% of dynamic rigidity and the reduction of approximately 10% of hysteresis at 70 °C, were obtained by replacing 33% of CB with CB/SHP and 66% of CB with CB/SSP, as if only half of the dimeric pyrrole compound was actually effective. To explain this finding, it could be, for example, hypothesized that only one of the pyrrole rings reacts with the carbon substrate. To confirm this hypothesis, further research should be performed.

#### 3.4.4. Tensile Properties

**Figure 9:**
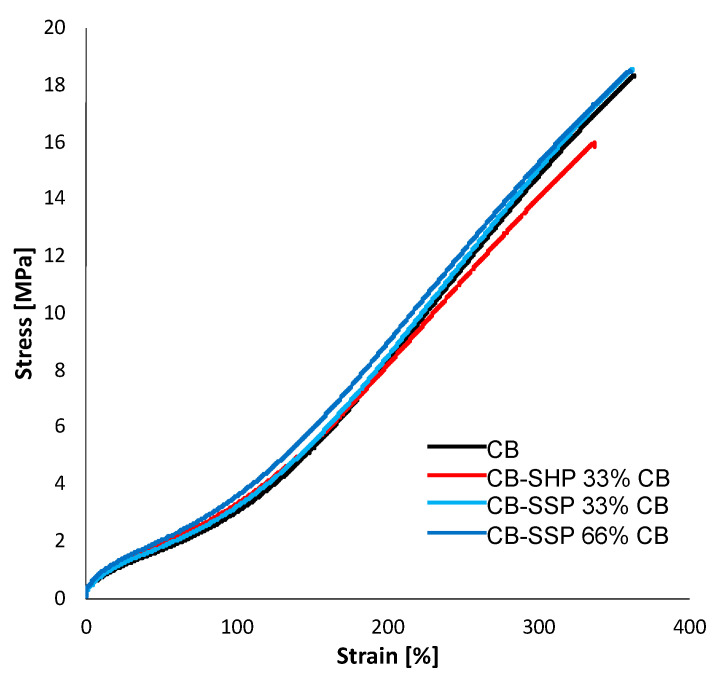
Tensile curves of -SBR/NR-based composites filled with CB or CB/CB/SHP or CB/CB/SSP.

Tensile properties were determined by means of quasi-static tensile tests. Results with corresponding standard deviations are reported in Table 5, while tensile curves are shown in Figure 9.

CB/PyC in place of CB gave rise to higher values of stresses at low strain (100% strain). CB/SSP brought about the increase of stresses at all the elongations together with better ultimate properties. The use of CB/SHP led to a decrease of stress at further elongations and to worse ultimate properties. The different ultimate properties, obtained with CB/SHP and CB/SSP, could be explained with a different filler dispersion, which could be worse in the presence of the former adduct. The thiol group is highly reactive and could promote a chemical bond between the filler and the elastomer chains already during the mixing, preventing the filler dispersion. This aspect was investigated and is discussed in the next paragraph. SHP as an ingredient did not lead to any enhancement of the static reinforcement at all the elongations and also at break.

#### 3.4.5. Filler Dispersion Analysis

**Figure 10:**
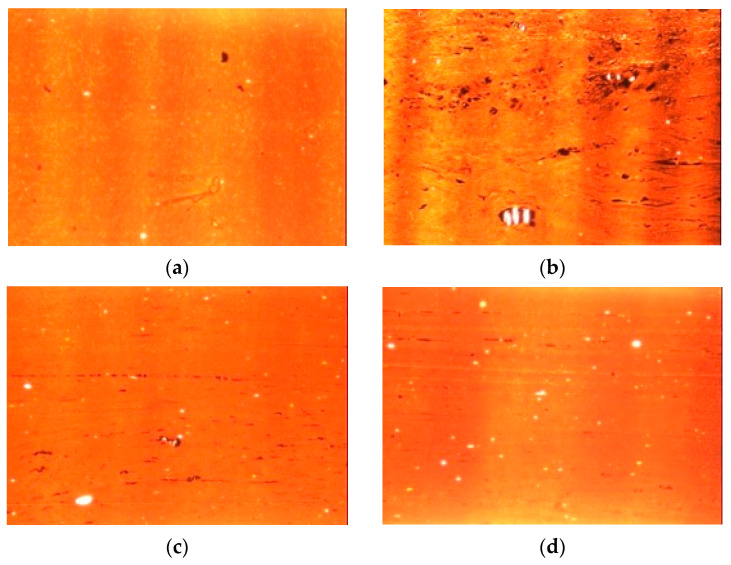
Microscopy images of the reference CB composite (**a**), the CB/SHP 33% CB composite (**b**), the CB/SSP 33% CB composite (**c**) and the CB/SSP 66% CB composite (**d**). The images are 771 × 576 micron.

A dispersion automated image analysis was carried out on the composite with CB as the only filler and on the composites with the adducts, both CB/SHP and CB/SSP. In Table 6, there are data about undispersed filler (%) and filler aggregates: number, minimum, maximum, and average diameter. Microscopy images are in Figure 10.

The comparison of Figure 10a,b reveals the worsening of the filler dispersion with the CB/SHP adduct in place of 33% of pristine CB. Instead, the images in Figure 10c,d highlight a good filler dispersion with CB/SSP in place of CB, both at 33% and 66% replacement. The percentage of undispersed filler and the size of the aggregates seem to be at least comparable to those of the reference composite. As a matter of fact, the composite with the greater amount of CB/SSP shows aggregates with a lower size, as if the dimeric pyrrole compound could promote the CB dispersion in the elastomer matrix.

### 3.5. Study of the Reactivity of SHP and SSP with Squalene

The results reported so far demonstrate that SHP and SSP behave as coupling agents for CB in elastomer composites crosslinked with a sulfur based system. They are bound to the CB surface through a covalent bond. This statement is supported not only by the prior art [43,44,45,46,47,48] but also by the results reported in this manuscript, namely the TGA data, particularly on the adducts after washing. The reactivity of the -SH and -SS- functional groups with the unsaturated elastomers could be reasonably assumed. However, to demonstrate that the pyrrole compound can establish a covalent bond between CB and the elastomer chains, we investigated the reactivity between either SHP or SSP and the unsaturated polymer chains by performing the reaction of the PyC with squalene, which is commonly used as the low-molar-mass homologue of natural rubber [61,62].

The reaction was performed at 150 °C for one hour under stirring at 200 rpm by adopting a PyC/squalene = 1/1 molar ratio, and aliquots of the reaction products were filtered and analyzed through atmospheric pressure chemical ionization mass spectrometry (APCI-MS). Moreover, the reaction of SHP with squalene was also performed in the presence of the vulcanization ingredients.

#### 3.5.1. Reactivity of SHP with Squalene

**Figure 11:**
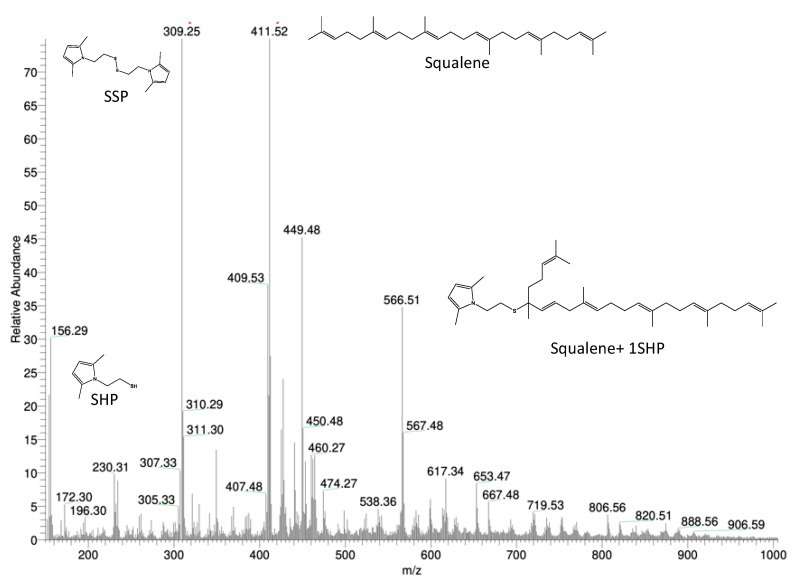
Mass spectrum of the product of the reaction between squalene and SHP.

The mass spectrum of the reaction product is shown in Figure 11, together with the structure of the chemical substances correlated with the most abundant peaks, which were attributed to: SHP, the SSP dimer, squalene and a product consisting of a deprotonated molecule of SHP bound to a molecule of squalene (C_30_H_49_-C_8_H_12_NS).

Since the expected molar mass of the latter product was 563.97 g/mol, the peak with molar mass 566.51 was fragmented to verify the nature of its related compound. The compound’s mass spectrum is shown in Figure S2 in the Supplementary Material. The fragmentation confirmed that the 566 g/mol peak compound corresponds to squalene with an added deprotonated SHP molecule, thus confirming the hypothesized chemical structure.

#### 3.5.2. Reactivity of SSP with Squalene

The mass spectrum of the reaction product is shown in Figure S3 in the Supplementary Material, together with the structure of the identified chemical substances. A compound made by a SHP radical moiety bound to a highly oxidized squalene was identified. It can be assumed that the split-up of the sulfur bridge in SSP is homolytic, and this leads to two radicals in SHP, which then react with squalene.

#### 3.5.3. Reactivity of SHP with Squalene in the Presence of the Vulcanization System

The reaction of SHP with squalene was also performed in the presence of the typical ingredients of a vulcanization system: ZnO, stearic acid, sulfur and N-tert-butyl-2-benzothiazyl sulfenamide. The recipes for the reaction mixture are in Table S2 in the Supplementary Material. A reference reaction, without SHP, was carried out.

The mass spectrum of the reference compound, shown in Figure S4 in the Supplementary Material, revealed the presence of squalene molecules linked by mono- and polysulfidic bridges as well as squalene molecules bearing thiols and sulfur atoms.

**Figure 12:**
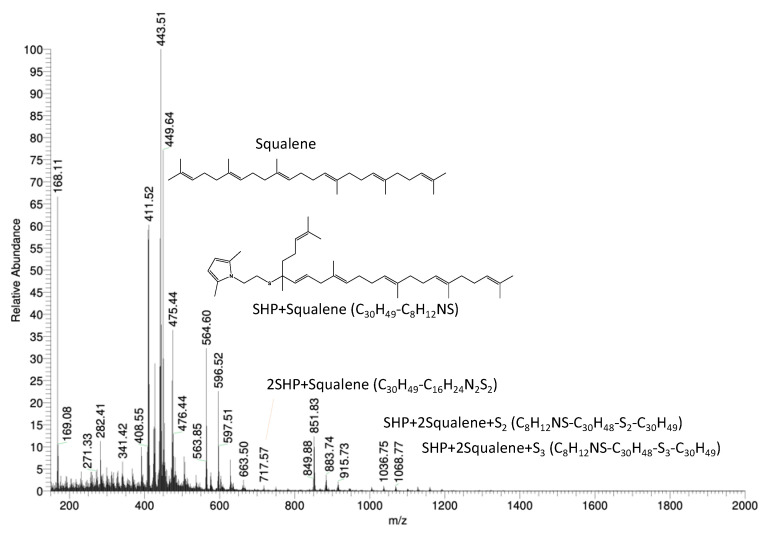
Mass spectrum of squalene-based SHP model compound.

The mass spectrum of the product of the reaction in the presence of SHP is shown in Figure 12, along with the chemical structure of the identified chemical compounds.

**Figure 13:**
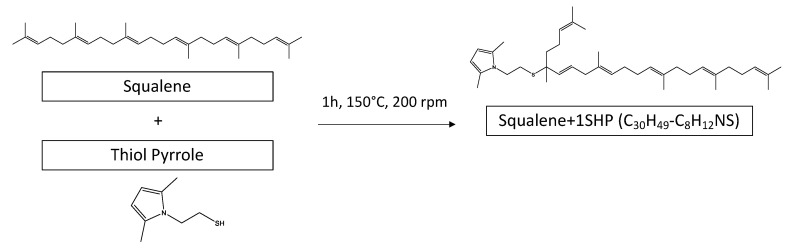
Reaction between SHP and squalene.

SHP was bound once or twice to a single squalene molecule or to squalene molecules linked by mono- or polysulfide chains. These findings indicate the ability of SHP to react with unsaturated substrates in the presence of a sulfur-based vulcanization system. The reaction of SHP with squalene can thus be represented as in Figure 13.

A radical is generated from SHP under heating and in the presence of oxygen. Then, the radical species formed from SHP reacts with squalene via the extraction of a hydrogen in the allylic position and the direct addition of a second molecule of the radical SHP derivative. This mechanism is in line with what has been reported in the literature [61,62,63]. In the case of SSP, first the opening of the -S-S- bridge and then the reaction of SHP occur.

## 4. Conclusions

The work reported here demonstrates that silica is not the only filler to be used for reducing the hysteresis of elastomer composites based on unsaturated elastomers. This objective was achieved by using a CB functionalized with either SHP or SSP, pyrrole compounds that are covalently bound onto the CB surface and contain sulfur based functional groups reactive with the elastomer chains. The replacement of even a minor part of CB (33%) with CB/SHP and of a larger amount of CB (66%) with CB/SSP resulted in an increase of dynamic rigidity (E′) of more than 20% and to a reduction in hysteresis of approximately 10% at 70 °C.

SHP and SSP were synthesized via one-pot two-step synthesis starting from biosourced chemicals, cysteine and cysteamine, achieving 78% and 73% yield, respectively. The CB/SHP and CB/SSP adducts were prepared by CB with the PyC, mixing, and heating, achieving high functionalization yields of 92% for the CB/SHP adduct and 97% for the CB/SSP adduct. The whole functionalization process was thus characterized by high carbon efficiency.

The composites were based on S-SBR and NR. In addition to the effect on the dynamic-mechanical properties mentioned above, better curing efficiency and similar, if not better, ultimate tensile properties, in particular with CB/SSP, were obtained. Even CB dispersion was achieved with SSP as the pyrrole compound. These results were not replicated by using SHP as an ingredient in the elastomer composite.

The reactivity of the pyrrole compounds with the unsaturated elastomer chains was demonstrated via the reaction with squalene as the model compound.

It is thus proven that SHP and SSP act as coupling agents for CB in the elastomer composites.

The best overall composite’s properties were achieved with the CB/SSP adduct, with a 66% replacement of pristine CB. The same dynamic properties were obtained with CB/SHP in place of 33% of CB. The hypothesis could be proposed that only one of the pyrrole rings reacts with the carbon substrate, and thus only half of the dimeric pyrrole compound is effective.

Such reactive carbon black could represent an alternative to the silica/silane systems. This work paves the way not only for the easy functionalization of CB but also for the use of pyrrole compounds as coupling agents, avoiding the need for further chemicals. The technologies involved allow for an easy scaling up for a large-scale application such as the one in tyre compounds.

## 5. Patents

A patent [53] resulted from the work reported in this paper.

## Figures and Tables

**Table 1:** S-SBR/NR-based composites with CB, CB and CB/SHP or CB and CB/SSP as reinforcing fillers ^a^.

Ingredient	CB	CB + SHP	CB/SHP 33% CB	CB/SSP 33% CB	CB/SSP 66% CB
S-SBR 4630	70	70	70	70	70
NR	30	30	30	30	30
CB	65	65	43.55	43.55	22.10
SHP		1.2			
CB/SHP	0	0	22.95	0	0
CB			21.45		
SHP			1.5		
CB/SSP	0	0	0	22.75	45.5
CB				21.45	42.9
SSP				1.3	2.6

^a^ Ingredient amounts expressed in phr; other ingredients added for each composite: stearic acid 2 phr, ZnO 2.5 phr, 6PPD 2 phr, TBBS 1.8 phr, PVI 0.5 phr, and sulfur 1.8 phr in the header of the table the amount of CB replaced is indicated in %.

**Table 2:** Mass losses of pristine CB, CB/SHP and CB/SSP determined via TGA analysis.

Sample		Mass Loss (%)			
		T < 150 °C	150 °C < T < 550 °C	550 °C < T < 900 °C	T > 900 °C (d)
CB	(a)	0.4	0.7	1.3	97.6
	(c)	0.3	0.1	0.1	99.5
CB/SHP	(b)	0.2	5.6	2.1	92.7
	(c)	0.03	5.2	1.9	93
CB/SSP	(b)	0.9	4.4	1.6	93
	(c)	0.6	4.2	1.6	95.4

(a) Pristine, (b) after the functionalization reaction, (c) after washing with acetone, and (d) includes residue, if present.

**Table 3:** Properties from the vulcanization of composites in Table 1.

Property	CB	CB + SHP	CB/SHP 33% CB	CB/SSP 33% CB	CB/SSP 66% CB
M_L_ (dNm)	3.6	3.6	4.0	3.9	4.2
M_H_ (dNm)	20.2	19.7	20.8	21.3	22.2
M_H_ − M_L_ (dNm)	16.6	16.1	16.8	17.5	18
t_S1_ (min)	3.3	2.4	3.1	3.1	3
t_90_ (min)	8.8	6.2	7.2	7.8	7.4
(M_H_ − M_L_)/(t_90_ − t_s1_) (dNm/min) ^a^	3.0	4.3	4.1	3.7	4.2

^a^ The curing rate was calculated by means of the following equation: $curing\;rate=\frac{M_{H}-M_{L}}{t_{90}-t_{s1}}$.

**Table 4:** Axial dynamic-mechanical properties of composites in Table 1.

Property	Temperature (°C)	CB	CB + SHP	CB-SHP 33% CB	CB-SSP 33% CB	CB-SSP 66% CB
E′ (MPa)	10	15.60	14.92	18.48	17.33	18.49
	23	11.64	11.21	13.94	12.95	13.92
	70	6.69	6.42	8.21	7.47	8.11
E″ (MPa)	10	9.06	8.88	9.77	9.63	9.90
	23	5.37	5.44	5.91	5.76	5.97
	70	1.61	1.71	1.83	1.75	1.82
Tan Delta	10	0.58	0.60	0.53	0.56	0.54
	23	0.46	0.49	0.42	0.44	0.43
	70	0.24	0.27	0.22	0.23	0.22
ΔE′ (E′@10 °C–E′@70 °C) (MPa)	/	8.91	8.49	10.27	10.16	9.86

**Table 5:** Tensile properties of composites in Table 1.

Property	CB	CB + SHP	CB/SHP 33% CB	CB/SSP 33% CB	CB/SSP 66% CB
σ_100_ (MPa)	3.05 ± 0.14	3.08 ± 0.03	3.33 ± 0.06	3.23 ± 0.16	3.60 ± 0.18
σ_200_ (MPa)	8.32 ± 0.46	7.90 ± 0.15	8.20 ± 0.01	8.51 ± 0.36	8.99 ± 0.35
σ_300_ (MPa)	14.84 ± 0.64	14.50 ± 0.19	14.07 ± 0.09	15.06 ± 0.33	15.28 ± 0.48
σ_B_ (MPa)	18.33 ± 1.30	18.42 ± 0.53	15.86 ± 0.68	18.55 ± 0.12	18.45 ± 0.28
ε_B_ (%)	363.18 ± 28.24	366.54 ± 6.06	337.18 ± 9.43	362.26 ± 5.05	360.73 ± 6.43
Energy at break (MJ/m^3^)	29.27 ± 4.60	29.22 ± 1.45	24.24 ± 1.65	29.77 ± 0.57	30.62 ± 0.30

**Table 6:** Results from the filler dispersion analysis on composites in Table 1.

Property	CB	CB/SHP 33% CB	CB/SSP 33% CB	CB/SSP 66% CB
Filler (%)	35.79	35.79	35.79	35.79
N° of aggregates	12	132	20	12
Min diameter (μm)	13	9	11	11
Max diameter (μm)	29	52	34	18
Average	20	18	20	15
Standard deviation	5	7	7	2
Undispersed filler (%)	0.2	1.5	0.3	0.2

## Data Availability

The data that support the findings of this study are available from the corresponding author upon reasonable request.

## Supplementary Materials

The following supporting information can be downloaded at: https://www.mdpi.com/article/10.3390/nano13202761/s1, Figure S1: Rheometric curves for rubber composites of Table 1. Figure S2: Reaction of SHP with squalene. Atmospheric pressure chemical ionization mass spectrometry on the reaction product. Fragmentation of the 563.97 g/mol compound. Figure S3: Reaction of SSP with squalene. Atmospheric pressure chemical ionization mass spectrometry on the reaction product. Mass spectrum zoom: 150–500 *m*/*z* (a) and 500–1450 *m*/*z* (b). Figure S4: Reaction of SHP with squalene in the presence of the vulcanization system. Mass spectrum of the reference reaction in the absence of SHP. Table S1: Shear dynamic-mechanical properties from strain-sweep experiments on composites of Table 1. Table S2: Reaction of SHP with squalene in the presence of the vulcanization system. Recipes in phr.
