# Supplementary material for: Bio-Based Pyrrole Compounds Containing Sulfur Atoms as Coupling Agents of Carbon Black with Unsaturated Elastomers
Source: Nanomaterials (Basel). 2023 Oct 14;13(20):2761. doi: 10.3390/nano13202761 (PMC10608980; doi:10.3390/nano13202761)
Supplement: Supplementary file 1 [file nanomaterials-13-02761-s001.zip › nanomaterials-2614614-supplementary.pdf]

Bio-Based Pyrrole Compounds Containing Sulfur Atoms as Coupling Agents of Carbon Black with Unsaturated Elastomers

Gea Prioglio [1], Simone Naddeo [1], Ulrich Giese, Vincenzina Barbera [1*] and Maurizio Stefano Galimberti [1,*]

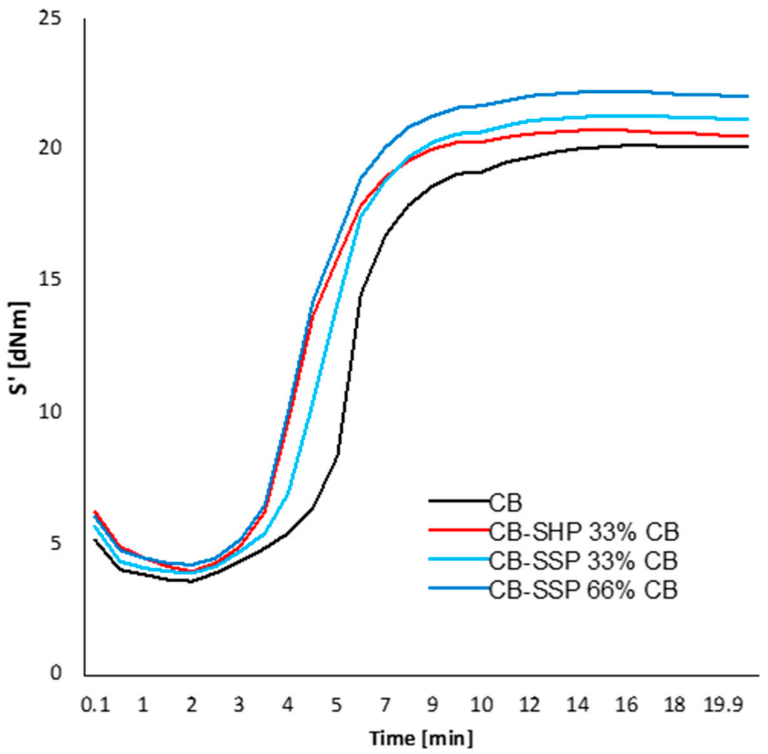

Figure S1. Rheometric curves for rubber composites of Table 1.

Table S.1. Shear dynamic-mechanical properties from strain-sweep experiments on composites of Table 1.

Property	CB	CB + SHP	CB/SHP 33% CB	CB/SSP 33% CB	CB/SSP 66% CB
$G'_{0.1\%}$ (MPa)	6.78	7.06	5.1	6.82	6.55
$G'_{25\%}$ (MPa)	1.26	1.32	1.36	1.37	1.43
$\Delta G' = \Delta(G'_{0.1\%} - G'_{25\%})$	5.52	5.75	4.64	5.45	5.12
$\Delta G'/ G'_{0.1\%}$	0.81	0.81	0.91	0.80	0.78
G''_{max} (MPa)	0.92	0.96	0.74	0.88	0.84
Tan $Delta_{max}$	0.34	0.33	0.28	0.31	0.29

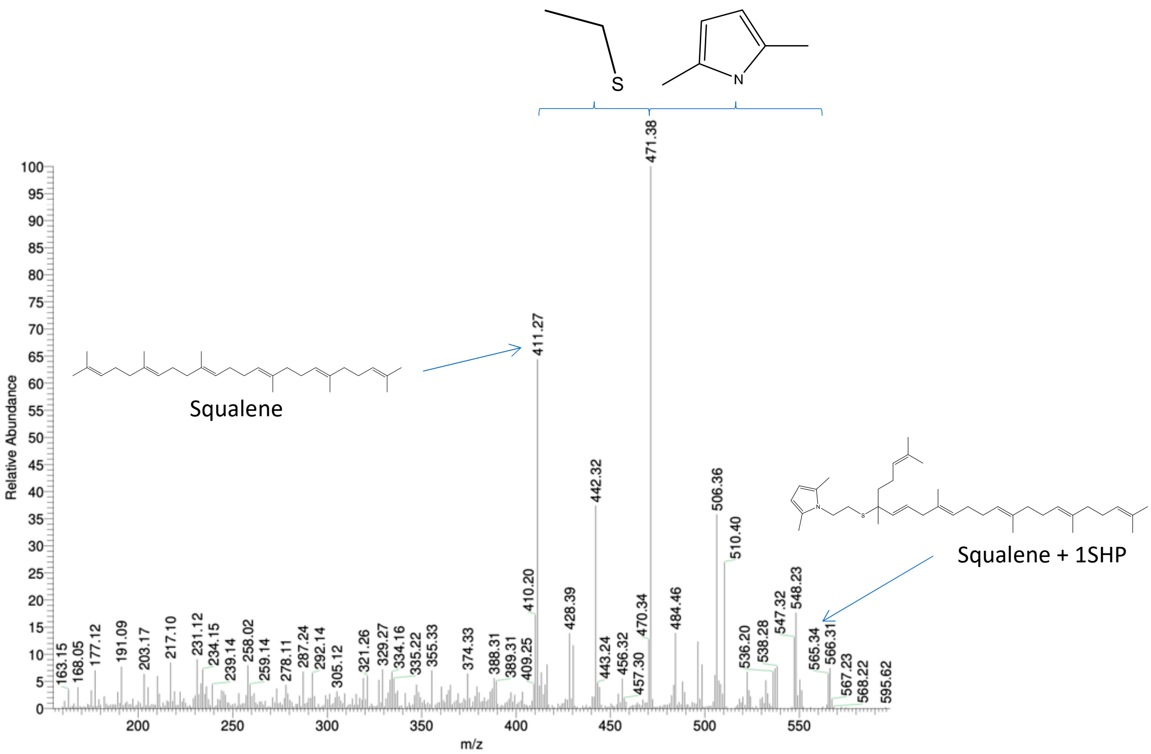

Figure S.2 Reaction of SHP with squalene. Atmospheric pressure chemical ionization mass spectrometry on the reaction product. Fragmentation of the 563.97 g/mol compound.

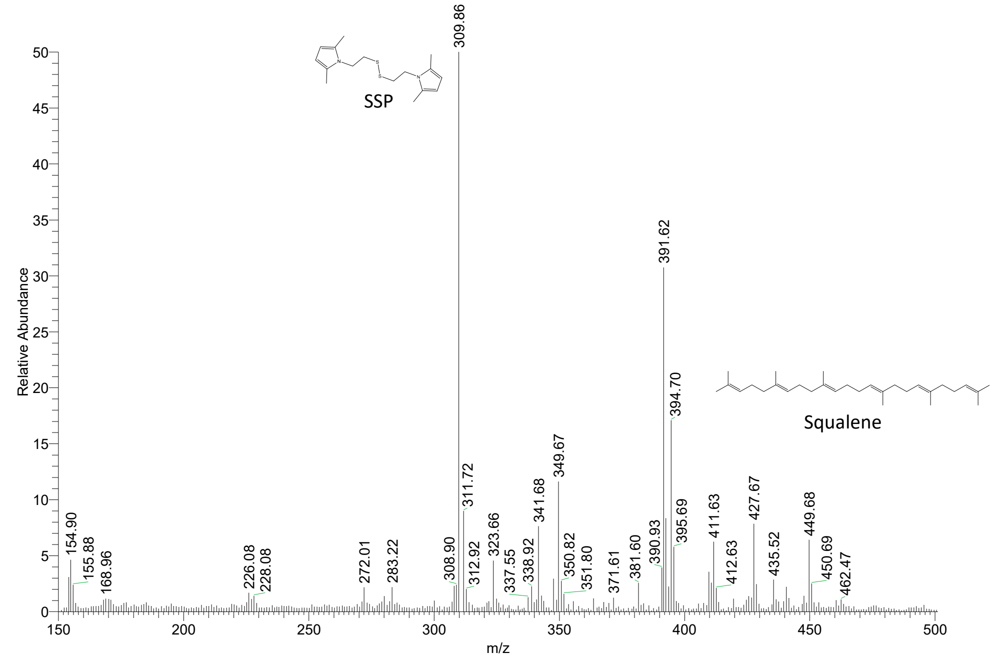

(a)

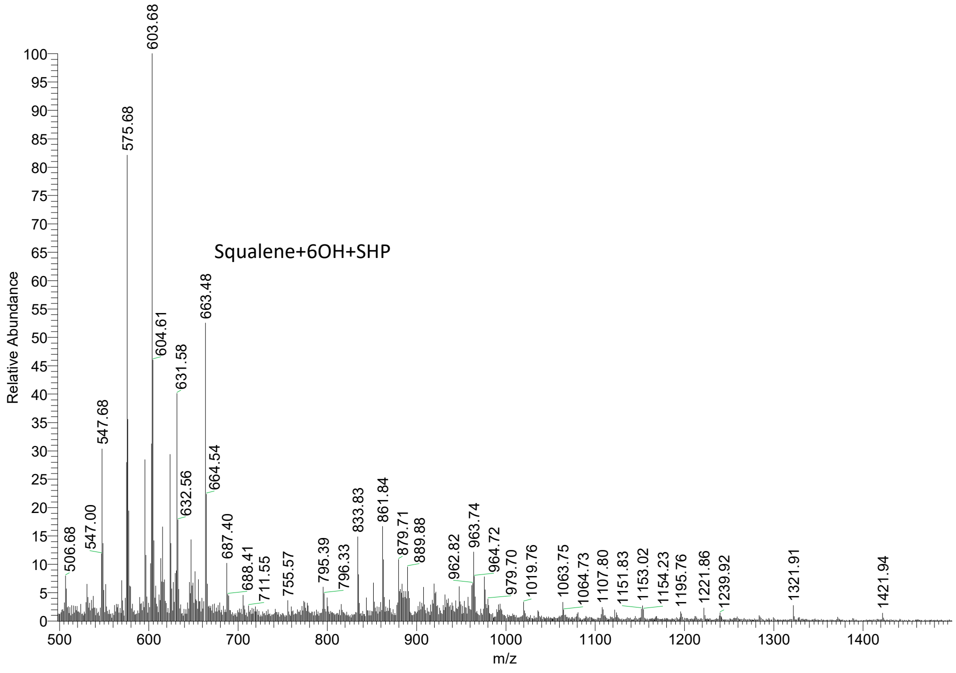

(b)

Figure S.3 Reaction of SSP with squalene. Atmospheric pressure chemical ionization mass spectrometry on the reaction product. Mass spectrum zoom: 150-500 m/z (a) and 500-1450 m/z (b)

Table S.2 Reaction of SHP with squalene in the presence of the vulcanization system. Recipes in phr

Ingredient	Reference[a]	SHP
Squalene	100	100
SHP	0	1.5
Stearic acid	2	2
Zinc oxide	2.5	2.5
TBBS	1.8	1.8
Sulphur	1.8	1.8

[a] reference reaction in the absence of SHP

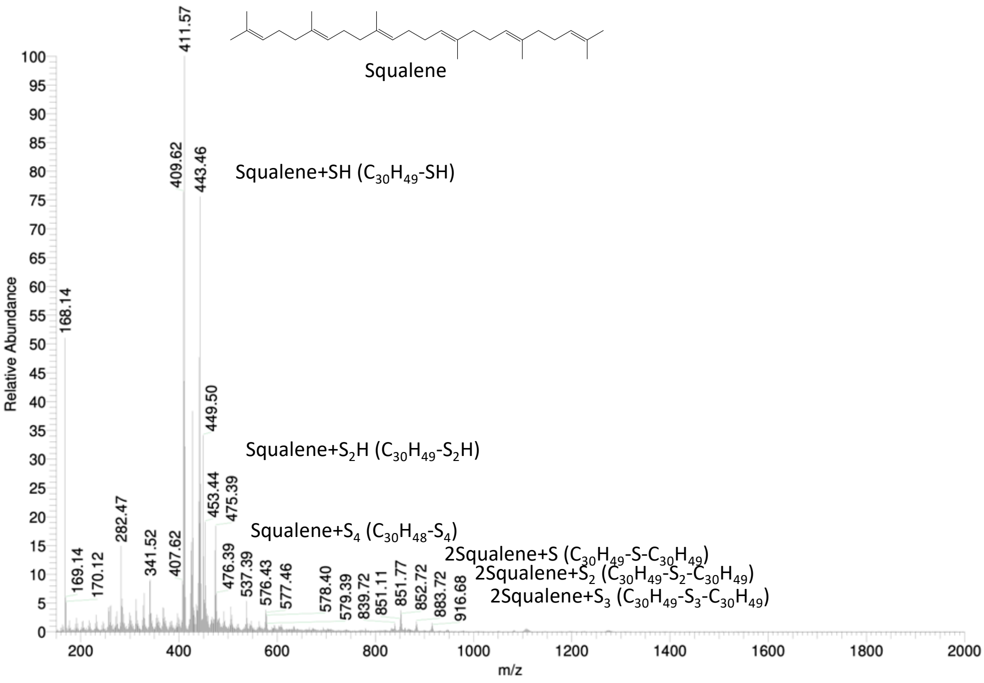

Figure S.4. Reaction of SHP with squalene in the presence of the vulcanization system. Mass spectrum of the reference reaction in the absence of SHP
